# Pathological subtype dictates survival and failure pattern in laryngeal neuroendocrine neoplasms: a 15-year single-institution experience

**DOI:** 10.1186/s12957-026-04506-0

**Published:** 2026-07-20

**Authors:** Feifei Qiao, Na Liu, He Li, Meng Zhang, Shubin Dong

**Affiliations:** 1 Department of Head and Neck Surgery, Shanxi Hospital Affiliated to Cancer Hospital, Cancer Hospital, Shanxi Provincial Cancer Hospital, Chinese Academy of Medical Sciences, Shanxi Medical University, Taiyuan, 031000 China; 2 Department of Anesthesiology, Shanxi Hospital Affiliated to Cancer Hospital, Cancer Hospital, Shanxi Provincial Cancer Hospital, Chinese Academy of Medical Sciences, Shanxi Medical University, Taiyuan, 031000 China; 3 Department of Pathology, Shanxi Hospital Affiliated to Cancer Hospital, Cancer Hospital, Shanxi Provincial Cancer Hospital, Chinese Academy of Medical Sciences, Shanxi Medical University, Taiyuan, 031000 China; 4 Department of Orthopedics, The Second Hospital of Shanxi Medical University, Taiyuan, 031000 China

**Keywords:** Laryngeal neuroendocrine neoplasms, Neuroendocrine carcinoma, Surgical treatment, Prognosis

## Abstract

**Background:**

Laryngeal neuroendocrine neoplasms (LNENs) comprise < 1% of laryngeal malignancies, and evidence-based treatment guidelines remain lacking. We sought to delineate clinicopathological features, therapeutic strategies, and survival determinants in a single-institution cohort of consecutive patients with primary LNEN.

**Methods:**

We retrospectively identified 31 consecutive patients with primary LNEN treated between 2010 and 2024. Histology was centrally reviewed by two head-and-neck pathologists and categorized as well-differentiated neuroendocrine tumor (NET, G1–G3) or poorly differentiated neuroendocrine carcinoma (NEC, small-cell/large-cell). All cases underwent immunohistochemistry (Syn, CgA, Ki-67) and multidisciplinary therapy. Survival analyses were performed with Kaplan–Meier and Cox proportional-hazards models.

**Results:**

Median age was 60 years, 90.3% were male, and 80.6% had a smoking history. NEC accounted for 71% (22/31) and NET for 29% (9/31). NEC exhibited higher Ki-67 index (85% vs. 10%, *P* < 0.001), more advanced stage (63.6% vs. 33.3%, *P* = 0.013), and a predilection for distant metastasis (85.7% vs. 25.0%, *P* = 0.002). Median overall survival (OS) was 27.0 months for NEC vs. not reached for NET (*P* < 0.001); 5-year OS was 0% vs. 44.4%. Median disease-free survival (DFS) was 19.0 vs. not reached (*P* < 0.001). Multivariate analysis identified NEC subtype (HR = 4.23, 95% CI 1.39–12.90), T3–4 stage (HR = 2.61, 95% CI 1.02–6.66), and lymph nodal positivity (HR = 3.12, 95% CI 1.20–8.10) as independent predictors of poor OS; NEC subtype (HR = 3.67, 95% CI 1.15–11.70) and N+ (HR = 3.51, 95% CI 1.22–10.10) predicted inferior DFS. Distant metastasis was the first mode of failure in 68.9% of curative-intent cases.

**Conclusions:**

Pathologic subtype (NEC vs. NET) emerges as the dominant independent predictor of survival in this cohort. NEC demonstrates a systemic biological phenotype characterized by early distant metastasis. These findings support the hypothesis that intensified perioperative systemic therapy, rather than locoregional escalation, warrants prospective investigation. Multi-center, molecularly stratified trials are needed to establish a precision-medicine paradigm for this rare disease.

**Supplementary Information:**

The online version contains supplementary material available at https://doi.org/10.1186/s12957-026-04506-0.

## Introduction

Laryngeal neuroendocrine neoplasms (LNENs) constitute fewer than 1% of all laryngeal malignancies and have an estimated annual incidence of 0.02–0.04 per million population [1, 2]. Population-based studies from North America and Europe report similar incidence rates, whereas Asian registries, including China, record even lower figures, underscoring the rarity of LNENs across all continents [3–5].Their extreme rarity has confined the literature to single-case reports or small retrospective cohorts of fewer than 15 patients; consequently, robust evidence regarding epidemiology, natural history, and optimal management is lacking [6, 7]. The 2022 WHO classification of head-and-neck tumors replaced the traditional “typical” and “atypical” carcinoid labels with a two-tier system: well-differentiated neuroendocrine tumors (NET, G1–G3) and poorly differentiated neuroendocrine carcinomas (NEC), subdivided into small-cell (SCNEC) and large-cell (LCNEC) variants [8]. Although the schema emphasises combined assessment of differentiation and proliferation, morphological and immunophenotypic overlap among primary laryngeal subtypes continues to hamper diagnostic reproducibility and complicate treatment decisions.

In routine clinical practice, the combined evaluation of morphology and immunohistochemistry (IHC) remains the gold standard for diagnosing LNENs and assigning grade [9]. Because cellular features, extent of necrosis and mitotic activity often form a continuous spectrum, routine haematoxylin–eosin(HE) sections alone are insufficient for precise subtyping. Expression of synaptophysin(Syn), chromogranin A(CgA) and CD56 confirms neuroendocrine differentiation, while the Ki-67 proliferation index in hot-spot areas defines the boundary between G1, G2 and G3 NET versus SCNEC/LCNEC [10]. These subtypes exhibit markedly different biology and prognosis: NEC progresses rapidly and can metastasise early, with a 5-year overall survival(OS) below 20%, whereas well-differentiated NET (G1) may be compatible with long-term survival and 5-year OS rates approaching 90% [9, 11]. Consequently, accurate pathological classification at presentation directly determines the intensity of subsequent multidisciplinary management, which ranges from local excision alone to combined chemoradiotherapy.

No uniform treatment protocol has been established for LNENs. For localised NET, radical surgery remains the mainstay; however, the confined anatomy of the larynx frequently forces a compromise between complete resection and organ preservation, and there is no consensus on when—or whether—to add post-operative radiotherapy. SCNEC/LCNEC is usually managed with platinum-etoposide chemotherapy ± radiotherapy extrapolated from pulmonary protocols, yet objective response rates, optimal number of cycles, and radiation fractionation schedules are based on small, heterogeneous case series. To date, no large, single-institution study has compared the outcomes of NET and NEC within a unified multidisciplinary framework. To address this gap, we conducted a 15-year retrospective analysis of 31 consecutive, pathologically confirmed LNENs, aiming to clarify their clinicopathological features, treatment strategies, and prognosis and to provide evidence for standardised management.

## Materials and methods

### Study design and patients

This single-center retrospective cohort study included consecutive patients with primary LNENs treated at Shanxi Cancer Hospital between January 1, 2010, and December 31, 2024. Inclusion criteria were: (1) histopathologically confirmed LNENs (NET G1–G3 or NEC); (2) primary tumor located in the larynx; (3) no concurrent extralaryngeal neuroendocrine tumors; and (4) complete medical records and follow-up data. Exclusion criteria included metastatic disease to the larynx, previous or synchronous malignancies, and incomplete data. This study was reviewed and approved by the Medical Ethics Committee of Shanxi Cancer Hospital (ethics approval number: KY2026-033), and informed consent has been duly obtained from both the patients and their families.

### Pathological review

Histopathological examination, including HE staining and IHC, remains the gold standard for diagnosing LNENs. On HE sections, architectural patterns such as organoid, nested, or diffuse infiltration, combined with cytologic features like high nuclear-to-cytoplasmic ratio and finely granular “salt-and-pepper” chromatin, raise the suspicion of neuroendocrine differentiation. Once suspected, confirmatory IHC is performed using a panel of neuroendocrine markers—including neuron-specific enolase (NSE), CgA, Syn, and CD56—along with epithelial markers such as cytokeratin (CK) and epithelial membrane antigen (EMA) to confirm epithelial origin.

The Ki-67 proliferation index was calculated in hotspot areas. Classification and grading were performed according to the 2022 WHO classification of head and neck tumors, supplemented by quantitative criteria established for gastroenteropancreatic neuroendocrine neoplasms: G1 (Ki-67 ≤ 5%), G2 (Ki-67 6–20%), and G3 (Ki-67 > 20%) for NET; NEC was diagnosed primarily by morphology (small-cell or large-cell features) with Ki-67 typically > 20%. Each case was systematically reassigned to one of the following categories: well-differentiated NET grade 1, 2, or 3, or poorly differentiated NEC, including small-cell and large-cell subtypes. All formalin-fixed paraffin-embedded (FFPE) tissue blocks and corresponding HE sections were independently re-evaluated by two senior head and neck pathologists (He Li and Jing Lian) with over 10 years of experience, under blinded conditions. Inter-pathologist concordance for the NET-versus-NEC distinction was 100% (31/31). Subtyping concordance within NEC was 90.9% (20/22): two cases originally reported as ‘poorly differentiated neuroendocrine carcinoma, NOS’ were reclassified as LCNEC (*n* = 1) and SCNEC (*n* = 1) after blinded review. All 9 NET cases remained concordant for grade (G1/G2).

### Pre-treatment evaluation and treatment planning

#### Imaging and endoscopic examination

All patients underwent contrast-enhanced neck CT/MRI for T/N staging and chest CT to exclude pulmonary metastasis. Abdominal imaging (ultrasound or contrast-enhanced CT) was performed to assess for liver or adrenal involvement. PET-CT was added selectively based on multidisciplinary team (MDT) decision. Indications for PET-CT included: (i) suspected distant metastasis or equivocal nodal disease on conventional CT/MRI; (ii) pre-operative staging of biopsy-proven NEC with high metastatic risk; or (iii) patient preference after cost counseling, as PET-CT was not uniformly reimbursed during the study period. In patients who did not receive PET-CT, staging was completed with contrast-enhanced CT alone, and no occult metastases were identified during follow-up. All patients also underwent digital laryngoscopy to assess tumor extent and vocal fold mobility. Endoscopic biopsy was performed during the procedure to obtain pre-operative histology when feasible.

#### Laboratory tests

Baseline laboratory tests included complete blood count, serum chemistry panel, and thyroid function profile. Anesthesia risk (ASA class) and nutritional status were documented preoperatively.

### MDT-based treatment strategy

All cases were presented at the weekly MDT meeting. Treatment decisions considered (1) pathologic subtype (NET vs. NEC), (2) TNM stage and tumor site, (3) proximity to critical laryngeal structures, and (4) patient age, ASA class, and preference. Strategies ranged from single-modality function-preserving surgery to combined treatment including total laryngectomy, neck dissection, chemotherapy and/or radiotherapy.

### Follow-up and endpoints

Patients were assessed at 4 weeks post-treatment, then every 3 months for 2 years, every 6 months for years 3–5, and annually thereafter. Each visit included digital laryngoscopy and neck ultrasound; contrast-enhanced neck CT was added every 6 months or whenever recurrence was suspected. Chest CT and abdominal imaging (CT or ultrasound) were performed annually or when clinically indicated. Patients unable to attend clinics were contacted by telephone to maintain follow-up continuity. All suspected relapses were verified histologically or by MDT consensus.

Overall survival (OS) was calculated uniformly from the date of histopathological diagnosis (surgical resection or diagnostic biopsy) to death from any cause or to 31 December 2025, whichever came first. Disease-free survival (DFS) was measured from the date of curative-intent surgery to the first evidence of tumor recurrence/metastasis or to 31 December 2025, whichever occurred first. The two non-surgical patients were included in OS but excluded from DFS analysis.

### Statistical analysis

All analyses were performed with R 4.3.0 and SPSS 27.0. Continuous data are presented as median (IQR) and categorical data as n (%). Group comparisons used the χ² or Fisher’s exact test for categorical variables and the Mann–Whitney U test for continuous variables.

OS and DFS were estimated using Kaplan–Meier curves and compared by the log-rank test. Competing risks (locoregional vs. distant failure) were analysed with cumulative incidence and Gray’s test. Cox models were built with forward–backward stepwise selection from variables showing *P* < 0.10 in the univariate analysis; proportional-hazards assumptions were verified using Schoenfeld residuals. To minimize overfitting in this rare-disease cohort, only variables with *P* < 0.10 on univariate analysis were eligible for multivariate entry. The resulting events-per-variable (EPV) ratios were 7.0 for OS (21 events / 3 variables) and 10.5 for DFS (21 events / 2 variables), satisfying conventional thresholds for stable effect estimation. HRs are reported with 95% CIs. All tests were two-sided; *P* < 0.05 was considered significant.

## Results

### Baseline characteristics

This single-center consecutive cohort enrolled 31 patients diagnosed with LNENs between January 2010 and December 2024, representing 0.6% (31/5167) of all laryngeal malignancies treated at our institution. Median age was 60 years (IQR 55–65) and 90.3% were male; 80.6% had a positive smoking history. The primary site was supraglottic in 67.7% of cases, glottic/subglottic in 32.3%.

According to the 2022 WHO classification, 71.0% (22/31) were NEC (14 SCNEC, 8 LCNEC) and 29.0% (9/31) were NET (7 G2, 2 G1; no G3 NET was identified). Within the NET subgroup, median Ki-67 index was 3% (IQR 2–5) for G1 and 15% (IQR 8–20) for G2. Median Ki-67 index was 80% (IQR 55–90): 85% (60–90) in NEC versus 10% (5–20) in NET (*P* < 0.001). Advanced stage (III–IV) was present in 54.8% (17/31) of patients, including 16 with locally advanced disease (stage III–IVA) and one with distant metastasis (stage IVC). The rate of cervical lymph-node metastasis was also 54.8% (17/31), with N2 disease in 82.4% (14/17) of these cases. Compared with NET, NEC lesions more often involved the glottic/subglottic region (40.9% vs. 11.1%, *P* = 0.016), presented at higher stage (63.6% vs. 33.3% III–IV, *P* = 0.013) and received adjuvant chemotherapy (59.1% vs. 0%, *P* < 0.001) and radiotherapy (77.3% vs. 11.1%, *P* < 0.001) more frequently. At last follow-up, mortality was 86.4% in NEC and 22.2% in NET (*P* < 0.001).Detailed baseline characteristics are provided in Table 1.

**Table 1:** Baseline characteristics of 31 patients with laryngeal neuroendocrine neoplasms

Characteristic	Total (*n* = 31)	NEC (*n* = 22)	NET (*n* = 9)	*P*
Age, n (%)				0.060
<60 year	13 (41.9)	10 (45.5)	3 (33.3)	
≥60 year	18 (58.1)	12 (54.5)	6 (66.7)	
Sex, n (%)				0.080
Female	3 (9.7)	2 (9.1)	1 (11.1)	
Male	28 (90.3)	20 (90.9)	8 (88.9)	
Smoking history, n (%)				0.021
No	6 (19.4)	3 (13.6)	3 (33.3)	
Yes	25 (80.6)	19 (86.4)	6 (66.7)	
Tumor location, n (%)				0.016
Supraglottic	21 (67.7)	13 (59.1)	8 (88.9)	
Glottic/subglottic	10 (32.3)	9 (40.9)	1 (11.1)	
AJCC stage, n (%)				0.013
I–II	14 (45.2)	8 (36.4)	6 (66.7)	
III–IV	17 (54.8)	14 (63.6)	3 (33.3)	
Ki-67 index, median (IQR)	80 (55–90)	85 (60–90)	10 (5–20)	< 0.001
Adjuvant chemotherapy, n (%)				< 0.001
No	18 (58.1)	9 (40.9)	9 (100.0)	
Yes	13 (41.9)	13 (59.1)	0 (0.0)	
Adjuvant radiotherapy, n (%)				< 0.001
No	13 (41.9)	5 (22.7)	8 (88.9)	
Yes	18 (58.1)	17 (77.3)	1 (11.1)	
Status at last follow-up, n (%)				< 0.001
Alive	10 (32.3)	3 (13.6)	7 (77.8)	
Dead	21 (67.7)	19 (86.4)	2 (22.2)	

### Treatment modalities

All 31 patients were evaluated by the head-and-neck MDT. Individualized plans were based on pathological grade, TNM stage, and functional preservation goals: NET-G1/G2 lesions received curative-intent, function-preserving surgery, whereas SCNEC/LCNEC were managed with multimodal therapy. Details are summarised in Table 2.

**Table 2:** Treatment modalities and short-term outcomes (*n* = 31)

Treatment	Total(*n* = 31)	NEC(*n* = 22)	NET(*n* = 9)
Primary site surgery	29(93.5%)	21(95.5%)	8(88.9%)
Function-preserving surgery	19(65.5%)	12(57.1%)	7(87.5%)
Total laryngectomy	10(34.5%)	9 (42.9%)	1(12.5%)
R0 resection	27(93.1%)	20 (95.2%)	7(87.5%)
Neck dissection	25(80.6%)	19(86.4%)	6(66.7%)
Unilateral	10(40.0%)	7(36.8%)	3(50.0%)
Bilateral	15(60.0%)	12(63.2%)	3(50.0%)
Median lymph nodes removed, median (range)	21(10–54)	22(12–54)	18(10–38)
Pathologically positive nodes	15(60.0%)	13(68.4%)	2(33.3%)
Radiotherapy	19(61.3%)	18(81.8%)	1(11.1%)
Post-operative adjuvant	18(94.7%)	17(94.4%)	1(100.0%)
Radical/palliative	1(5.3%)	1(5.6%)	0(0.0%)
Chemotherapy	15(48.4%)	14(63.6%)	1(11.1%)
Trimodality(S + R+C)	13(41.9%)	13(59.1%)	0(0.0%)
Successful decannulation(≤6 mo)†	13/15(86.7%)	10/12(83.3%)	3/3(100.0%)

*S* Surgery, *R* Radiotherapy, *C* Chemotherapy

†Only patients who underwent function-preserving surgery and required temporary tracheostomy were included

Twenty-nine patients (93.5%) underwent tumor resection. Function-preserving surgery was performed in 19 cases (65.5%): CO₂-laser transoral resection (*n* = 3), endoscopic excision (*n* = 1), supraglottic horizontal partial laryngectomy (*n* = 12) and vertical partial laryngectomy (*n* = 3). Total laryngectomy was required in 10 patients (34.5%), usually for T3–T4 glottic/subglottic NEC. An R0 resection was achieved in 93.1% (27/29); the two patients with positive margins after CO₂-laser resection underwent prompt re-excision.

Therapeutic neck dissection was carried out in all 16 cN+ patients (unilateral 5, bilateral 11). Of 13 cN0 cases, 9 underwent elective levels II–III dissection, 6 of whom had NEC. Overall, 25/31 patients (80.6%) had neck dissection with a median of 21 nodes removed (range 10–54); 60.0% (15/25) were pathologically positive.

Adjuvant therapy was guided by pathology and risk factors–defined as T3–4 stage, nodal positivity (N+), positive resection margin, or NEC subtype.Thirteen patients (44.8%) received concurrent chemoradiotherapy—all had NEC and ≥ 1 high-risk feature. Five patients (17.2%) were given radiotherapy alone (four elderly/ASA ≥ III NEC, one pN2c NET-G2). Eleven patients (37.9%) received no adjuvant treatment: four low-risk NEC (three pT1–2N0; one T3N2bM0 declined because of complications) and seven pT1–2N0 NET-G1/G2. Consequently, adjuvant radiotherapy was administered to 62.1% (18/29) and adjuvant chemotherapy to 44.8% (13/29) of operated cases.

Two patients (6.5%) were managed without primary surgery: one stage IVc patient received systemic chemotherapy only; one 85-year-old ASA III patient opted for definitive intensity-modulated radiotherapy plus chemotherapy.

Functional outcomes were recorded in 15 patients who required temporary tracheostomy during organ-preserving surgery. Thirteen (86.7%) were successfully decannulated within 6 months; two required 11 months because of radiation-induced edema. All total-laryngectomy patients had a permanent tracheostomy created without peri-operative mortality.

### Survival outcomes

Median follow-up was 38 months (IQR 24–61; range 10–164). All 31 patients completed at least 10 months of post-treatment follow-up. All patients were included; 29 received curative-intent therapy and 2 non-surgical treatment. 

#### Overall survival

**Fig. 1:**
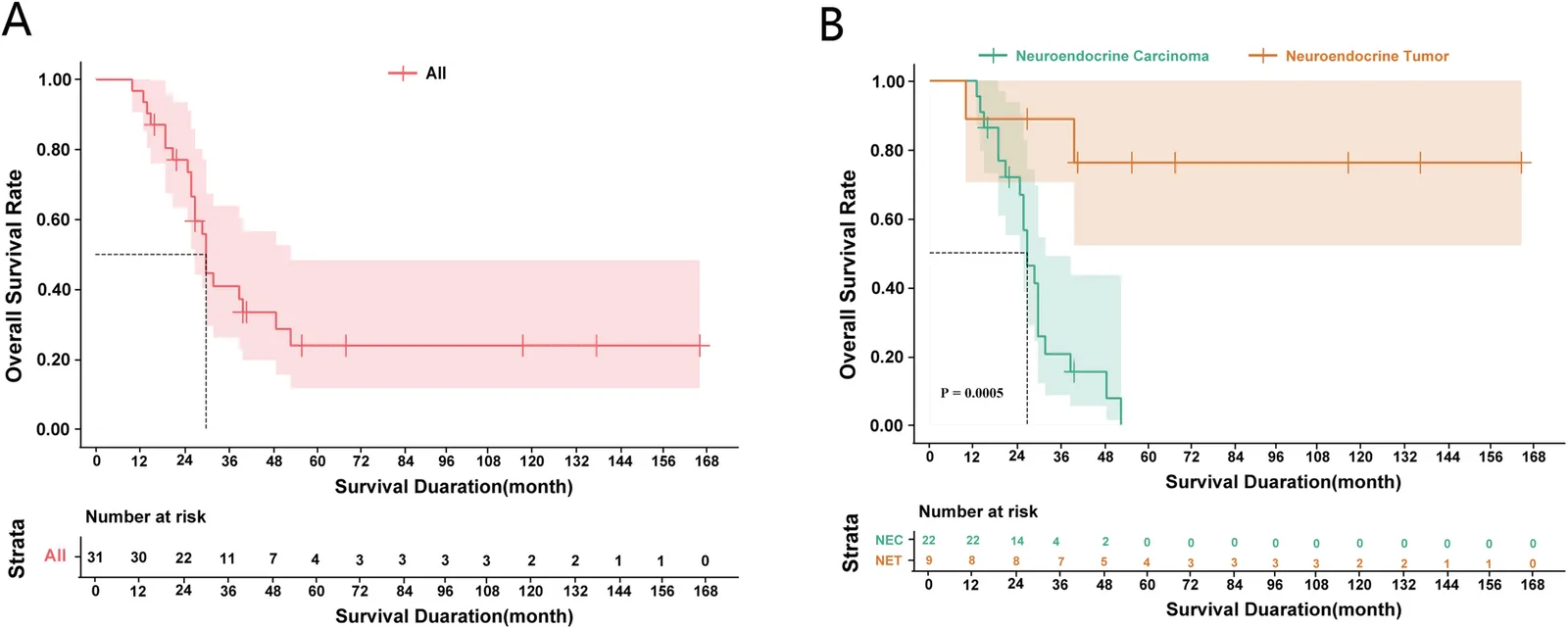
Overall survival in laryngeal neuroendocrine neoplasms. **A** Kaplan–Meier estimate of OS for the entire cohort (n=31). Median OS was 30.0 months (95% CI, 27–53). **B** OS stratified by pathological subtype. NEC (n=22) showed significantly inferior survival compared with NET (n=9), with median OS of 27.0 months (95% CI, 25–32) versus not reached (*P* < 0.001)

At the time of last follow-up, 21 patients had died and 10 remained alive (2 with disease, 8 disease-free). Median OS was 30.0 months (95% CI 27–53) with 1-, 3- and 5-year survival rates of 96.8%, 35.5% and 12.9%, respectively (Fig. 1A).

By pathological subtype, NEC (*n* = 22) showed median OS 27.0 months (95% CI 25–32) and the 1 -, 3 -, and 5-year survival rates were 100.0%, 18.2%, and 0.0%. NET(*n* = 9) did not reach median OS and 1 -, 3 -, and 5-year survival rates were 88.9%, 77.8%, and 44.4%. The difference between the two groups was statistically significant (*P* < 0.001, Fig. 1B).

**Fig. 2:**
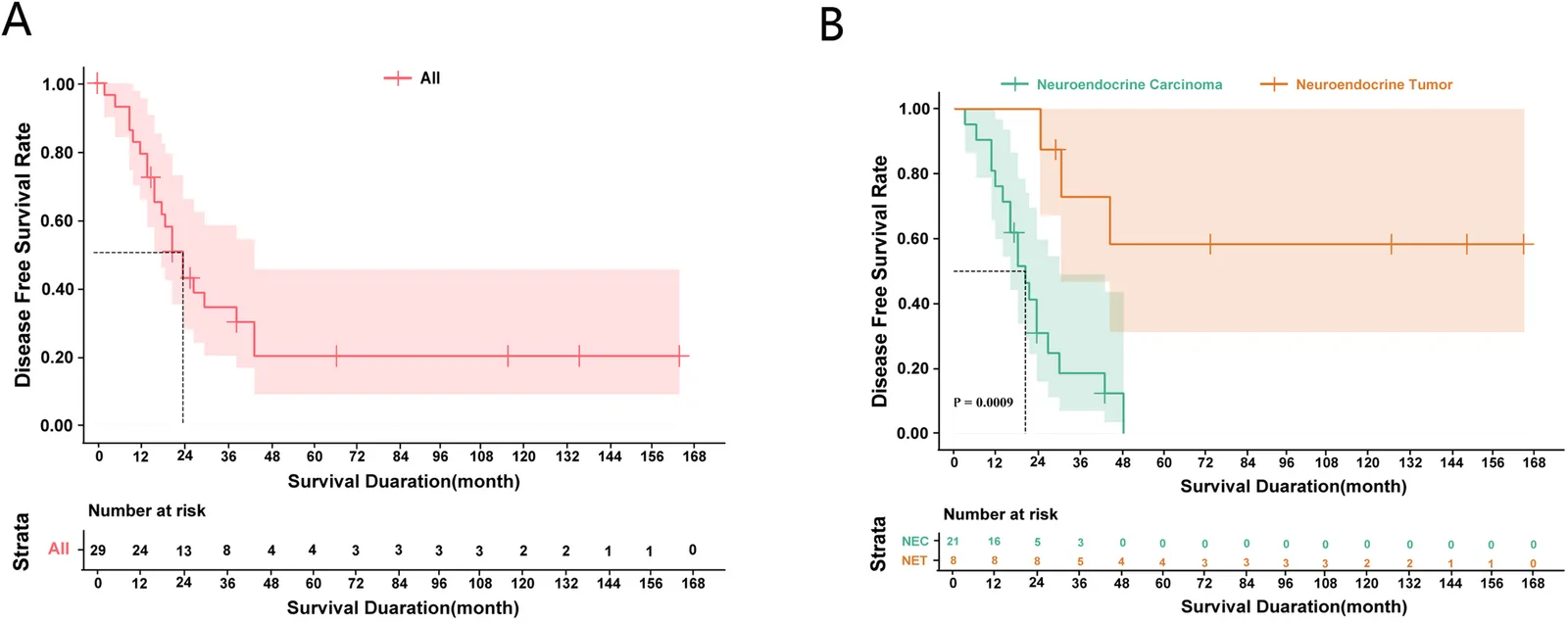
Disease-free survival (DFS) after curative-intent treatment. **A** Kaplan–Meier estimate of DFS for patients undergoing curative-intent surgery (n=29). Median DFS was 25.0 months (95% CI, 17–45). **B** DFS comparison between NEC (n=21) and NET (n=8) subgroups. Median DFS was 19.0 months (95% CI, 15–28) versus not reached, respectively (P < 0.001)

Disease-Free Survival. At the time of last follow-up, 21 of 29 patients (72.4%) who received curative-intent treatment had developed recurrence or metastasis. Median DFS was 25.0 months (95% CI 17–45), with 1-, 3- and 5-year DFS rates of 82.8%, 27.6% and 13.8%, respectively (Fig. 2A).

By pathological subtype, NEC (*n* = 21) had a median DFS of 19.0 months (95% CI 15–28) and 1-, 3- and 5-year DFS rates of 76.2%, 14.3% and 0.0%, respectively. NET (*n* = 8) did not reach median DFS; the corresponding 1-, 3- and 5-year DFS rates were 100.0%, 62.5% and 50.0%. The difference between the two groups was statistically significant (*P* < 0.001, Fig. 2B).

#### Early vs. locally advanced disease

To evaluate the impact of AJCC stage on survival, the 29 curative-intent patients were grouped into early-stage (I–II, *n* = 14) and locally advanced (III–IVA, *n* = 15) categories, and their OS and DFS were compared.

**Fig. 3:**
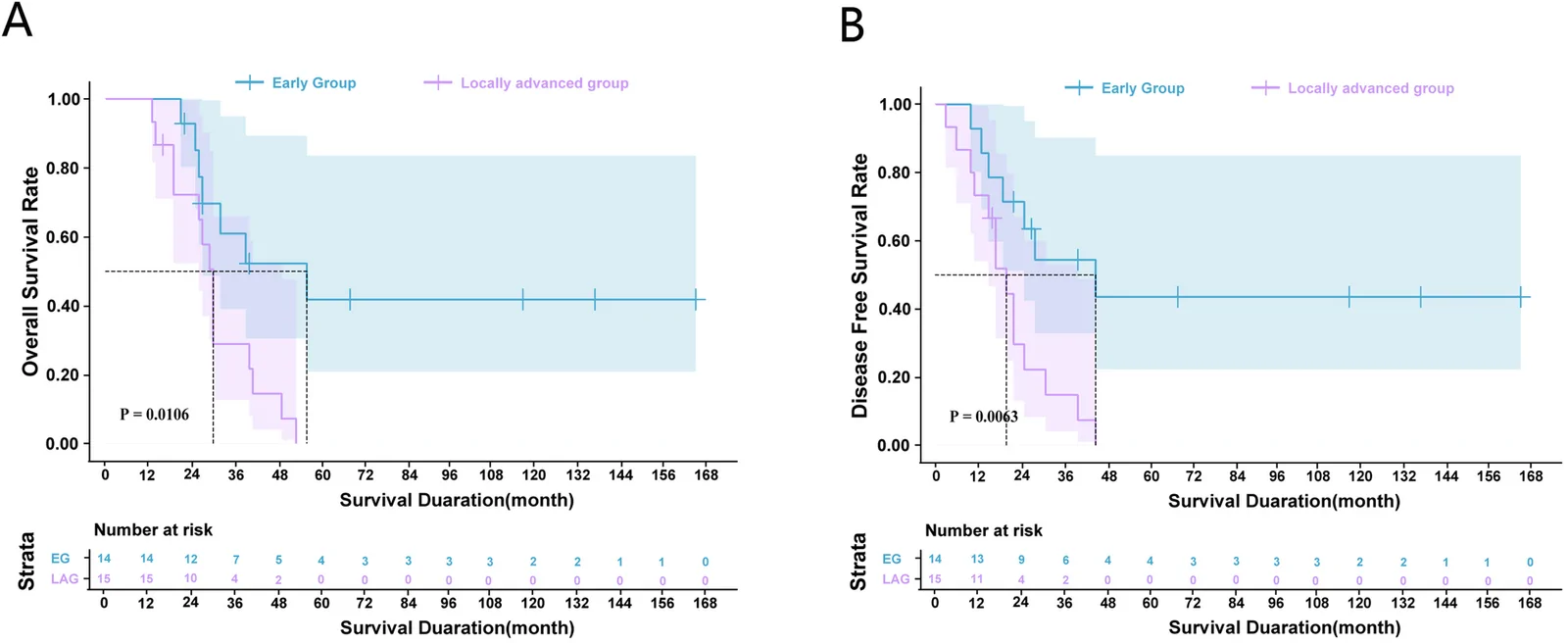
Survival outcomes by disease stage (early versus locally advanced). **A** Overall survival comparison between early-stage (I–II, n=14) and locally advanced (III–IVA, n=15) disease. Median OS was 56.0 months versus 30.0 months (P = 0.011). **B** Disease-free survival comparison between early-stage and locally advanced disease. Median DFS was 45.0 months versus 20.0 months (P = 0.006)

**Fig. 4:**
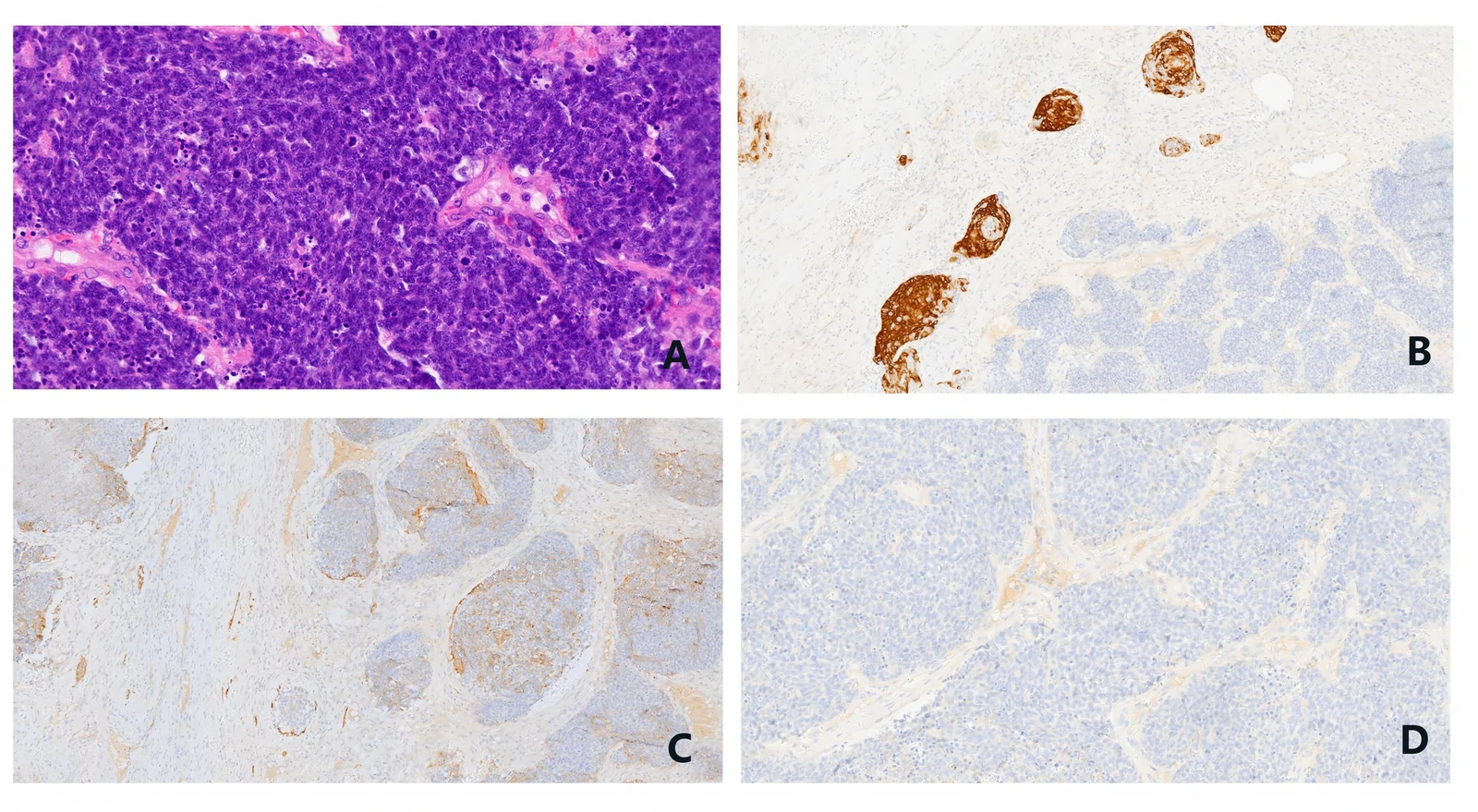
Representative histopathology and immunohistochemistry of well-differentiated neuroendocrine tumor (NET-G2). **A** Hematoxylin–eosin (HE) staining showing organoid nesting pattern with uniform nuclei and finely granular chromatin (×40). **B** Immunohistochemistry demonstrating CK5 positivity (×40). (C) Synaptophysin (Syn) positivity (×40). (D) Chromogranin A (CgA) positivity (×40)

**Fig. 5:**
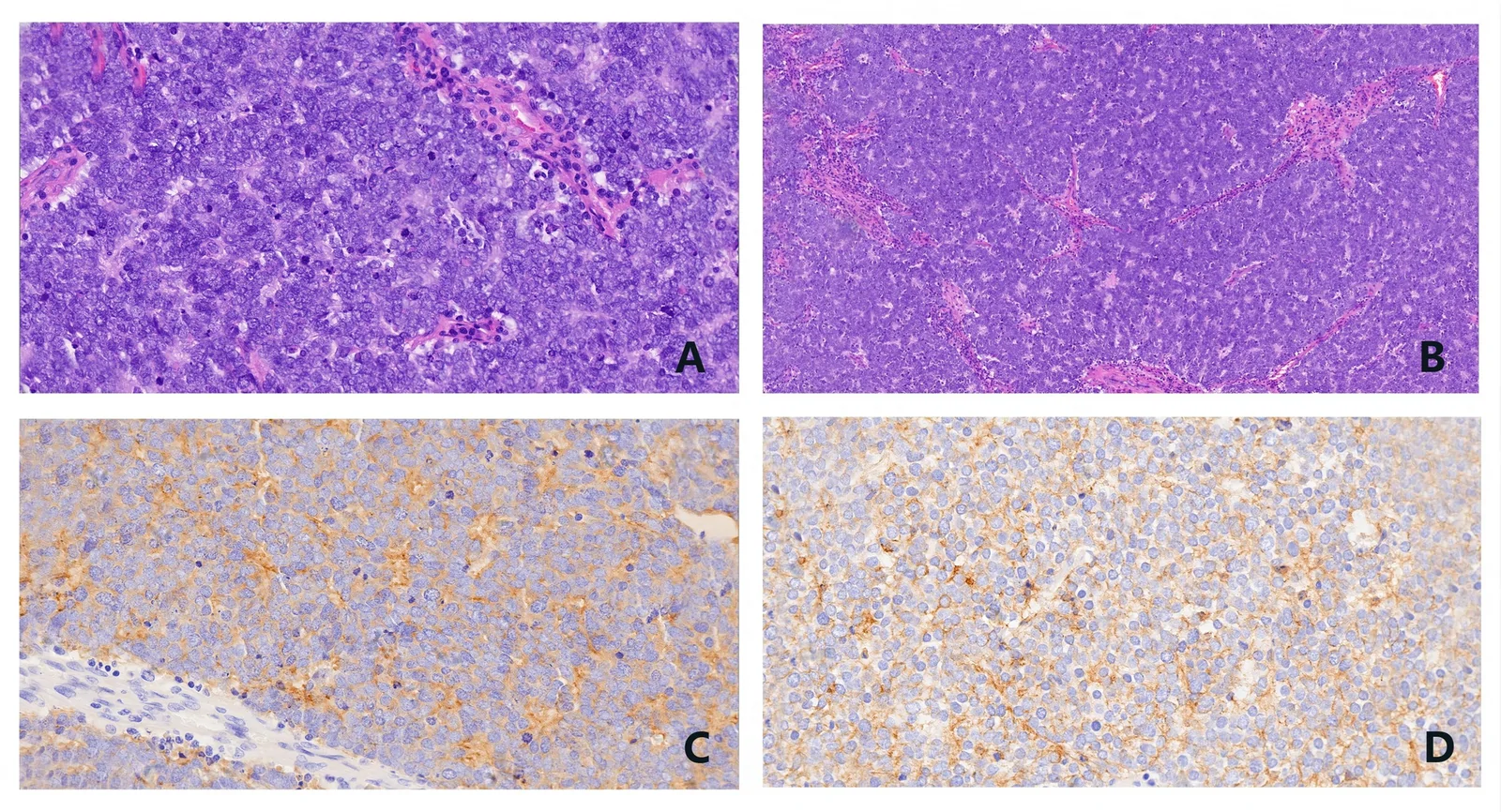
Representative histopathology and immunohistochemistry of small-cell neuroendocrine carcinoma (SCNEC). **A** HE staining showing diffuse sheets of small cells with scant cytoplasm, nuclear molding, and high nuclear-to-cytoplasmic ratio (×40). **B** Low-power HE view demonstrating tumor architecture with fibrovascular septa (×10). (C) Synaptophysin (Syn) positivity (×40). (D) CD56 positivity (×40)

Early-group (EG) patients had a median OS of 56.0 months (95% CI 27–not reached) and 1-, 3- and 5-year OS rates of 100.0%, 50.0% and 28.6%, respectively. The locally advanced group(LAG)showed a median OS of 30.0 months (95% CI 26–49) with corresponding OS rates of 100.0%, 26.7% and 0.0%. The difference was statistically significant (*P* = 0.011, Fig. 3A). Figs. 4 and 5.

Median DFS in the EG was 45.0 months (95% CI 25–not reached) and 1-, 3- and 5-year DFS rates were 92.9%, 42.9% and 28.6%. In the LAG median DFS was 20.0 months (95% CI 15–40) with DFS rates of 73.3%, 13.3% and 0.0%. This difference was also statistically significant (*P* = 0.006, Fig. 3B).

### Prognostic factor analysis

Univariate and multivariate Cox models were used to identify independent predictors of OS in all 31 patients and of DFS in the 29 who underwent curative-intent surgery. Variables with *P* < 0.10 on univariate analysis entered the multivariate model.

Overall survival.

Univariate analysis showed that T3–4 stage, nodal positivity and NEC subtype were significantly associated with shorter OS (Table 3). Multivariate analysis confirmed that T3–4 stage (HR = 2.61, 95% CI 1.02–6.66, *P* = 0.045), N+ (HR = 3.12, 95% CI 1.20–8.10, *P* = 0.020) and NEC subtype (HR = 4.23, 95% CI 1.39–12.90, *P* = 0.011) were independent adverse prognostic factors for OS (Table 3).

**Table 3:** Univariate and multivariate cox regression analysis for overall survival (*n* = 31)

Variable	Univariate		Multivariate	
	HR (95% CI)	*P* value	HR (95% CI)	*P* value
Age				
< 60	Ref			
≥ 60	1.21 (0.52–2.81)	0.662	—	—
Sex				
Female	Ref			
Male	1.05 (0.32–3.48)	0.935	—	—
Smoking history				
No	Ref			
Yes	1.38 (0.50–3.83	0.533	—	—
Tumor location				
Glottic/Subglottic	Ref			
Supraglottic	1.16 (0.49–2.72)	0.734	—	—
T classification				
T1–2	Ref			
T3–4	2.84 (1.15–7.02)	0.024	2.61 (1.02–6.66)	0.045
N classification				
N0	Ref			
N+	3.35 (1.31–8.57)	0.011	3.12 (1.20–8.10)	0.020
Histological subtype				
NET	Ref			
NEC	4.91 (1.67–14.4)	0.003	4.23 (1.39–12.9)	0.011
Adjuvant chemotherapy				
No	Ref			
Yes	0.85(0.43–2.29)	0.911	—	—
Adjuvant radiotherapy				
No	Ref			
Yes	0.89 (0.37–2.16)	0.798	—	—

*HR* Hazard ratio, *CI* Confidence interval, — Not entered into the final model

#### Disease-free survival

Among the 29 patients who underwent curative-intent treatment, multivariate analysis identified N+ (HR = 3.51, 95% CI 1.22–10.1, *P* = 0.022) and NEC subtype (HR = 3.67, 95% CI 1.15–11.7, *P* = 0.028) as independent predictors of DFS (Table 4).

**Table 4:** Univariate and multivariate cox regression analysis for disease-free survival after radical surgery (*n* = 29)

Variable	Univariate		Multivariate	
	HR (95% CI)	*P* value	HR (95% CI)	*P* value
Age				
< 60	Ref			
≥ 60	1.10 (0.45–2.68)	0.842	—	—
Sex				
Female	Ref			
Male	0.96 (0.25–3.68)	0.951	—	—
Smoking history				
No	Ref			
Yes	1.25 (0.42–3.71)	0.687	—	—
Tumor location				
Glottic/Subglottic	Ref			
Supraglottic	1.09 (0.44–2.70)	0.856	—	—
T classification				
T1–2	Ref			
T3–4	2.39 (0.94–6.08)	0.068	2.20 (0.85–5.69)	0.106
N classification				
N0	Ref			
N+	3.88 (1.39–10.8)	0.010	3.51 (1.22–10.1)	0.022
Histological subtype				
NET	Ref			
NEC	4.05 (1.31–12.5)	0.015	3.67 (1.15–11.7)	0.028
Adjuvant chemotherapy				
No	Ref			
Yes	0.92 (0.36–2.35)	0.861	—	—
Adjuvant radiotherapy				
No	Ref			
Yes	0.88 (0.34–2.27)	0.793	—	—

*HR* Hazard ratio, *CI* Confidence interval, — Not entered into the final model

### Pattern of failure

Among the 29 patients who received curative-intent therapy, distant metastasis was the predominant mode of first failure (68.9%, 20/29). Median time to any failure was 19.0 months (IQR 15–28.5) after surgery.

The rate of distant metastasis was significantly higher in NEC than in NET (85.7% vs. 25.0%, *P* = 0.002), as was the rate of any failure (85.7% vs. 37.5%, *P* = 0.020); median time to failure was shorter in NEC (*P* = 0.028).

Local recurrence occurred in 4 patients (13.8%), regional nodal relapse in 9 (31.0%), and 9 patients (31.0%) had simultaneous failure in ≥ 2 sites. Detailed patterns are summarised in Table 5.

**Table 5:** Pattern of first failure after curative-intent treatment (*n* = 29)

Failure site	Total(*n* = 29)	NEC(*n* = 21)	NET(*n* = 8)	*P* value*
Any failure	21 (72.4)	18 (85.7)	3 (37.5)	0.020
Local recurrence	4 (13.8)	4 (19.0 )	0 (0.0 )	0.289
Regional nodes metastasis	9 (31.0)	7 (33.3 )	2 (25.9 )	0.688
Distant metastasis	20 (68.9)	18 (85.7)	2 (25.0)	0.002
Simultaneous ≥ 2 sites	9 (31.0)	8 (38.1)	1 (12.5)	0.273
Median time to failure mo(IQR)	19.0(15.0−28.5)	18.0(14.0–27.0)	28.0(25.0–45.0)	0.028†

†Log-rank test

*Fisher’s exact test, NEC vs. NET

## Discussion

LNENs account for < 1% of laryngeal malignancies yet constitute the most aggressive non-squamous subgroup [1]. Compared with the far more common squamous cell carcinoma, LNENs exhibit markedly different biology: NEC—representing two-thirds of cases in our cohort—frequently metastasizes early, with a 5-year overall survival of < 20% [2, 12]. At presentation more than half of patients already harbour cervical lymph-node or distant metastases [7, 13, 14]. The present single-institution, 15-year consecutive series captures 31 histologically verified LNENs (0.6% of all laryngeal cancers treated). NEC predominated (71.0%, two-thirds SCNEC), whereas well-differentiated NET comprised 29.0% (mainly G2). Representative histopathological and immunohistochemical features of NET-G2 and SCNEC are illustrated in Fig. 4 and 5, respectively. Managed within a uniform multidisciplinary pathway, NEC patients achieved a median OS of only 27 months and a 3-year survival rate of 18.2%, compared with 77.8% for NET. By three years, 85.7% of NEC patients had suffered first failure—predominantly pulmonary or hepatic distant metastasis—underscoring the inadequacy of traditional larynx-centred “surgery ± radiotherapy” for this systemic disease. These data are consistent with the meta-analysis of 436 cases by van der Laan et al. [11]and provide contemporary, real-world evidence that histological subtype is the dominant survival determinant, mandating biology-driven treatment intensification.

Consistent with population-based data [1], our cohort shows a striking male predominance (90.3%) and close association with tobacco use (80.6%), with a median age at diagnosis of 60 years. The supraglottis—especially the epiglottis and ary-epiglottic folds—was the preferred site (67.7%), supporting previous series [9, 15, 16]. Notably, 88.9% of NET lesions arose in the supraglottis, whereas both subglottic tumors were LCNEC, suggesting an anatomical predilection that may reflect differing embryological origins or exposure patterns. Symptoms (sore throat, hoarseness, foreign-body sensation) mimic those of squamous carcinoma, but LNENs more often present as submucosal or exophytic masses. Over half of patients were stage III–IV at diagnosis (54.8%), with N2 disease in 82.4% of node-positive cases, underscoring the aggressive biology and rapid progression characteristic of this neoplasm. The median Ki-67 index was 80% (range 3–90%); 70.9% of cases exhibited ≥ 55% positivity, indicating universally high proliferation across our cohort.

These epidemiological and anatomical characteristics align closely with SEER and European registry data, supporting the external validity of our cohort demographics [3, 4, 11]. Nevertheless, treatment patterns—particularly the high rate of function-preserving partial laryngectomy for NET—may reflect institutional surgical expertise and regional practice preferences. Direct extrapolation to Western cohorts, where definitive chemoradiotherapy is more commonly employed for locally advanced disease, should therefore be cautious. Multi-center, multinational validation is needed to confirm whether our proposed pathology-first triage model is broadly applicable.

NEC comprised 71% of our cohort, with SCNEC representing 63.6% of all NEC cases, mirroring the epidemiological distribution described by Strojan et al. [12] and the MD Anderson series [7]. On multivariate analysis the NEC subtype remained the strongest independent predictor of both OS (HR = 4.23) and DFS (HR = 3.67), outweighing the influence of T or N stage. Only two NET-G1 cases were available (22.2% of the NET group), and no G3 NET was identified; our study is therefore underpowered to distinguish the indolent biology of G1 tumors from the potentially more aggressive behavior of G3 NET, which may share phenotypic overlap with NEC. This small sample size precludes reliable estimation of survival curves for G1-specific outcomes and limits our ability to establish Ki-67 thresholds predictive of metastatic risk within well-differentiated tumors. Consequently, our NET vs. NEC dichotomy, while clinically practical, may oversimplify the biological continuum of laryngeal neuroendocrine neoplasms. Multi-institutional collaborations are therefore required to fully characterise the biological continuum of LNENs.

Most prior studies of head-and-neck NENs are small heterogeneous series that mix multiple sites, limiting comparability. Built on a 15-year consecutive cohort, our study is the first to apply WHO-2022 criteria uniformly within a single-center MDT framework, compare failure patterns and survival between NET and NEC under a standardised treatment pathway, and represents one of the largest single-institution LNEN series to date. Sousa et al. [7] identified only 15 HG-NEC patients (13 laryngeal) at MD Anderson over 23 years; median OS was 17.8 months, virtually identical to our NEC subgroup. In that study 80% of radically treated patients relapsed, with mDFS 13.1 months, mirroring the 85.7% failure rate and 19-month mDFS observed here, reaffirming the systemic nature of laryngeal NEC. Notably, that series reported an objective response rate of approximately 50% to platinum–etoposide and a median progression-free survival of only 3.1 months, indicating insufficient depth and durability of conventional chemotherapy for laryngeal NEC. Strategies proven in small-cell lung cancer, such as immunochemotherapy or maintenance targeted therapy, are therefore urgently needed to improve long-term outcomes in patients with laryngeal NEC. Historically, treatment has been pathology-driven rather than function-sparing, with total laryngectomy rates of 30–60% and adjuvant radiotherapy in 60–80% of cases, but with wide variation in indications and timing [7, 17]. Employing a pathology-stage-function triage model, we achieved an R0 resection rate of 93.1% while limiting total laryngectomy to 34.5% of cases. Among patients undergoing organ-preserving surgery, 86.7% were successfully decannulated within 6 months without peri-operative mortality. However, this organ-preservation rate likely reflects selection bias inherent to our institutional referral patterns: patients with bulky glottic/subglottic tumors (40.9% of NEC) were more likely to undergo total laryngectomy, whereas supraglottic lesions amenable to horizontal partial laryngectomy predominated in the NET cohort. We acknowledge that treatment decisions were MDT-based and non-standardized, introducing selection bias that limits causal inference regarding the comparative efficacy of specific therapeutic strategies (e.g., surgery versus chemoradiotherapy). The observed outcomes reflect real-world, pragmatic decision-making rather than randomized comparisons. Thus, while function-preserving surgery appears feasible in selected patients, these results should not be generalized to all NEC cases, particularly those with subglottic extension or impaired vocal cord mobility.

The dominant first-failure pattern in NEC was distant metastasis (85.7% vs. 25.0% in NET, *P* = 0.002), primarily to the lung and liver, a phenotype reminiscent of extrapulmonary small-cell carcinoma. Our observed failure pattern aligns closely with the MD Anderson experience reported by Sousa et al. [7], where 80% of radically treated patients experienced relapse with a mDFS of only 13.1 months. In our cohort, 85.7% of NEC patients developed recurrence with a median DFS of 19.0 months. This concordance across independent centers supports the systemic nature of laryngeal NEC, distinct from the locoregional predominance seen in squamous cell carcinoma. However, unlike pulmonary NEC where TP53/RB1 co-loss drives early metastasis [18, 19], the molecular underpinnings of laryngeal NEC remain undefined due to limited genomic data, representing a critical gap for future investigation. We therefore speculate that laryngeal NEC shares comparable driver events—cell-cycle dysregulation and aberrant neuro-endocrine programming—that fuel rapid proliferation and metastasis [20]. The significantly shorter median time to relapse in NEC (18 vs. 28 months in NET) further underlines this aggressiveness. Going forward, we will employ high-depth next-generation sequencing to delineate the mutational landscape, copy-number alterations and immune microenvironment of laryngeal NEC, enabling rational testing of peri-operative systemic strategies such as EP combined with immune-checkpoint inhibitors, DLL3-targeted therapy or PRRT [7, 21].

On the basis of these findings we hypothesize that a pathology-first MDT protocol may be beneficial. For suspected laryngeal neuroendocrine neoplasms, biopsy with mandatory pathological and IHC review stratifies patients into NEC or NET as the first decision node. Patients with a pre-operative NEC diagnosis may be regarded as having systemic disease and offered intensive multi-modal therapy; adjuvant radiotherapy may be considered after surgery for locally advanced or N+ disease [20, 22].Conversely, NET-G1/G2 should be treated primarily with radical, function-preserving surgery; adjuvant radiotherapy is reserved for T3–T4 or N+ tumors to avoid over-treatment. However, these recommendations are hypothesis-generating and derive from a single-center, non-randomised study with limited numbers (only two G1 NET). Prospective, multicentre, molecularly stratified trials are therefore required to validate whether intensified peri-operative systemic therapy can reduce distant failure and prolong survival in laryngeal NEC.

This study has several limitations. First, its single-center retrospective design, despite consecutive enrolment and blinded pathological review, remains subject to selection bias, particularly regarding the feasibility of organ-preserving surgery in our cohort. Second, the small sample size (*n* = 31), while one of the largest single-institution LNEN cohorts reported, limits the precision of multivariate estimates; we therefore restricted the Cox model to key pre-specified variables and emphasize that all prognostic associations should be considered hypothesis-generating. Third, histological heterogeneity within the NET cohort represents an important limitation: with only two G1 cases (22.2% of the NET group) and no G3 NET identified, our study is underpowered to distinguish the indolent biology of G1 tumors from the potentially more aggressive behavior of G3 NET, which may share phenotypic overlap with NEC. This small sample size precludes reliable estimation of survival curves for G1-specific outcomes and limits our ability to establish Ki-67 thresholds predictive of metastatic risk within well-differentiated tumors. Consequently, our “NET vs NEC” dichotomy, while clinically practical, may oversimplify the biological continuum of laryngeal neuroendocrine neoplasms. Fourth, the long accrual period (2010–2024) could introduce treatment-evolution bias, as systemic therapy regimens evolved during this interval, though our institutional protocol remained relatively consistent. Finally, next-generation sequencing has not yet been performed, so associations between key mutations (TP53, RB1) and prognosis remain unexplored. Future multi-institutional registries should specifically stratify outcomes by G1/G2/G3 classification to validate optimal treatment de-escalation strategies for low-grade lesions.

## Conclusion

In this single-institution, consecutive, real-world cohort, pathological subtype (NEC vs. NET) emerged as the dominant independent predictor of OS and DFS. NEC exhibits a systemic phenotype with distant metastasis as the predominant pattern of first failure; these findings support the hypothesis that intensifying peri-operative systemic therapy, rather than locoregional escalation alone, may be critical for improving outcomes in laryngeal NEC; this hypothesis warrants validation in prospective, multi-center trials. 

## Supplementary Information

Supplementary Material 1.

## Data Availability

The datasets analyzed during the current study are available from the corresponding author on reasonable request.
